# Clinical and Neuroimaging Features of Tactile Hallucinations in Patients With Dementia With Lewy Bodies: A Cross‐Sectional Study

**DOI:** 10.1111/psyg.70082

**Published:** 2025-08-12

**Authors:** Kyosuke Kakeda, Hideki Kanemoto, Kenji Yoshiyama, Takashi Suehiro, Sumiyo Umeda, Yoshitaka Nakatani, Fuyuki Koizumi, Yuto Satake, Maki Yamakawa, Yuki Yamamoto, Kayako Isohashi, Hiroki Kato, Noriyuki Tomiyama, Mamoru Hashimoto, Manabu Ikeda

**Affiliations:** ^1^ Department of Psychiatry The University of Osaka Graduate School of Medicine Suita Osaka Japan; ^2^ Department of Psychiatry Kyoto Narabigaoka Hospital Kyoto Kyoto Japan; ^3^ Health and Counselling Centre The University of Osaka Toyonaka Osaka Japan; ^4^ Department of Psychiatry and Mental Health Sumitomo Hospital Osaka Osaka Japan; ^5^ Department of Psychiatry Osaka Psychiatric Medical Centre Hirakata Osaka Japan; ^6^ Department of Psychiatry Osaka General Medical Centre Osaka Osaka Japan; ^7^ Department of Psychiatry Minoh Neuropsychiatric Sanatorium Minoh Osaka Japan; ^8^ Brain Function Centre Nippon Life Hospital Osaka Osaka Japan; ^9^ Department of Diagnostic and Interventional Radiology The University of Osaka Graduate School of Medicine Suita Osaka Japan; ^10^ Department of Neuropsychiatry Kindai University Faculty of Medicine Osaka Sayama Osaka Japan

**Keywords:** cerebral blood flow, Lewy body dementia, magnetic resonance imaging, postcentral gyrus, tactile hallucinations

## Abstract

**Background:**

We examined the clinical features of tactile hallucinations associated with dementia with Lewy bodies and investigated their neural substrates.

**Methods:**

This retrospective observational cross‐sectional study enrolled 147 patients with probable dementia with Lewy bodies from the Department of Psychiatry, The University of Osaka Hospital, and collected the data of Clinical Dementia Rating, Mini‐Mental State Examination, Neuropsychiatric Inventory‐plus, and their demographics. Furthermore, magnetic resonance imaging and cerebral blood flow data from single‐photon emission computed tomography were collected when possible.

**Results:**

Ten patients (6.9%) had tactile hallucinations. There were no significant differences in age, sex, or cognitive function between the patients with and without tactile hallucinations. The patients with tactile hallucinations had more severe neuropsychiatric symptoms, although none developed delusional infestations. Magnetic resonance imaging showed lower grey matter volume in the right postcentral gyrus, and single‐photon emission computed tomography images showed lower cerebral blood flow in the left postcentral gyrus and relatively higher cerebral blood flow in the left inferior frontal gyrus in patients with tactile hallucinations than in those without. Single‐photon emission computed tomography was available for only three patients among those with tactile hallucinations. Cerebral blood flow in the postcentral gyrus was reduced in all three patients compared with the database of age‐sex‐matched healthy controls.

**Conclusions:**

These findings suggest that tactile hallucinations in patients with dementia with Lewy bodies may be related to damage in the postcentral gyrus and preserved function in the frontal lobe; this could be consistent with the “release phenomena” hypothesis of hallucinations.

## 1 Introduction

Dementia with Lewy bodies (DLB) is the second leading cause of neurodegenerative dementia among older people after Alzheimer's disease [1, 2]. Patients with DLB frequently exhibit neuropsychiatric symptoms (NPS) even in the early stages of the disease, and the severities of various NPS during the early stage are higher in patients with DLB than in those with Alzheimer's disease [3]. There are many cases of psychiatric‐onset DLB, a condition that is diagnosed as a psychiatric disorder before the patient presents with dementia [4], and many of these patients have highly systematized psychosis as the initial symptom [5]. NPS in the patients with DLB cause lower quality of life and a higher degree of caregiver distress [6, 7]. Therefore, NPS intervention is crucial for patients with DLB.

Among the various NPS associated with DLB, visual hallucinations (VH) are the most specific, and VH presence is one of the core clinical features in the diagnostic criteria [8]. There have been numerous studies on VH in DLB, including several studies on their neural basis [9, 10, 11]. Hallucinations other than VH can also occur in patients with DLB. A previous study reported that 35.5% of patients with DLB had auditory hallucinations, which were frequently accompanied by VH, similar to the background soundtracks of hallucinatory scenes [12]. Tactile hallucinations (TH) have also been observed in patients with Parkinson's disease (PD) [13, 14, 15] and DLB [16]. TH are symptoms of concern that are associated with intractable clinical entities such as delusional infestation [17] and cenesthopathy. However, previously published articles on TH in patients with PD and DLB are limited to case reports.

In the present study, we examined the prevalence and the features of TH associated with DLB and their neural substrates.

## 2 Methods

### 2.1 Study Design

This retrospective observational cross‐sectional study assessed general medical information and did not require interventions. For our analyses, we used data from the database of neuropsychological clinics and the Department of Psychiatry at The University of Osaka Hospital. All patients underwent standard neuropsychological examinations and routine laboratory tests. Their demographic information, including disease and drug history, were assessed through clinical interviews with their caregivers. Magnetic resonance imaging (MRI) of the brain and/or single‐photon emission computed tomography (SPECT) was also obtained, except in cases of infeasibility. All patient information was anonymised and stored as unlinked data prior to analysis to prevent the disclosure of personal information. No monetary incentives were provided to the participants or caregivers.

This study was approved by the Internal Review Board of The University of Osaka Graduate School of Medicine (No. 19117) and conducted in compliance with national legislation and the Declaration of Helsinki. All the patients and their families agreed to an opt‐out clause for the use of data collected during common clinical examinations.

### 2.2 Participants

Patients with mild cognitive impairment (MCI) [18] or dementia assumed to be due to Lewy bodies were retrospectively recruited according to the following criteria with reference to the consensus diagnostic criteria for probable DLB [8] and the research criteria for probable MCI with Lewy bodies [4] from the database of the neuropsychological clinic of the department of psychiatry in The University of Osaka Hospital between May 2005 and March 2020: (a) age ≥ 50 years old, (b) Clinical Dementia Rating (CDR) [19] score ≥ 0.5, (c) two or more core clinical DLB features (fluctuating cognition, VH, Parkinsonism and rapid eye movement sleep behavior disorder) present with or without the presence of indicative biomarkers, or only one core clinical feature present with positive results in one or more indicative biomarkers, and (d) reliable information about NPS obtained from family caregivers by the method described in the next subsection (Assessment of clinical features).

The exclusion criteria were: abnormal findings other than brain atrophy on MRI; complications or dementia history other than DLB; psychiatric diseases with onset before age 40 years that cause hallucinations or delusions; physical disorders known to affect brain function; severe complications of cardiovascular, hepatic, renal, or other diseases; and absence of a close caregiver who could state patient symptoms.

### 2.3 Assessment of Clinical Features

General cognition was assessed using the Mini‐Mental State Examination [20] and dementia severity was rated using the CDR. The NPS was assessed using the Japanese version of the Neuropsychiatric Inventory (NPI)‐plus [21], which includes the subitems of the original NPI‐12 (delusions, hallucinations, agitation/aggression, dysphoria, anxiety, euphoria, apathy, disinhibition, irritability/lability, aberrant motor behavior, sleep disturbances, and appetite/eating changes) [22] and subitems for cognitive fluctuation. According to the NPI‐12 methodology, we tabulated the types of delusions (persecution, theft, jealousy, phantom boarder, Capgras, home misidentification, abandonment, television sign, and others) and hallucinations (visual, auditory, conversational, tactile, olfactory, gustatory, and others). The presence or absence of TH was determined based on the results from the hallucination sub‐items in the NPI.

### 2.4 MRI Acquisition and Preprocessing

For brain imaging analysis, MRI of the following conditions was collected, if available, and performed within 3 months of the clinical assessments described above. Examinations were performed using a 1.5 Tesla MR system (SIGNA Explorer, General Electric, Milwaukee, WI, USA). Three‐dimensional volumetric acquisition of a T1‐weighted gradient‐echo sequence (3D‐T1) was conducted to produce a gapless series of thin sagittal sections covering the entire calvarium. The operating parameters were: field of view = 240 mm, matrix = 256 × 256, 124 × 1.40 mm contiguous sections, TR = 12.55 ms, TE = 4.20 ms, and flip angle = 15°.

MRI preprocessing was performed prior to statistical analysis using the Statistical Parametric Mapping (SPM) version 12b software (Wellcome Centre for Human Neuroimaging, UCL, London, UK) running MATLAB R2015a (The MathWorks Inc., Natrick, MA, US). All MRIs were segmented and transformed into a standard stereotaxic anatomical space with the brain template from the Montreal Neurological Institute Talairach and Tournoux atlas space (voxel size: 1.5 mm × 1.5 mm × 1.5 mm) using DARTEL flow created in the DARTEL process and smoothed using an 8‐mm Gaussian kernel.

### 2.5 SPECT Imaging Acquisition

For brain imaging analysis, SPECT images of the following conditions were collected, if available, within 3 months of the clinical assessments described above. The participants underwent N‐isopropyl‐p‐[123I]iodoamphetamine (123I‐IMP) SPECT using an integrated SPECT/CT system (Symbia T‐6 until February 2020, and Symbia Intevo. from March 2020; Siemens Healthineers, Erlangen, Germany) to evaluate regional cerebral blood flow (rCBF). The studies were performed with patients in a resting state with their eyes closed and ears unplugged. A 167‐MBq dose of 123I‐IMP was continuously injected into the antecubital vein. The SPECT scan was initiated 15 min after 123I‐IMP injection. Data acquisition was performed for 30 min in the static mode with circularly rotating gamma cameras over a 360° range in 4° angular steps (90 views) and with 150 s/cycle and 12 repeats. The radius of rotation was 17 cm. The scatter component of the projection data was estimated using the triple energy window method. Scatter‐subtracted tomographic data were reconstructed by the filtered‐back projection method with Chang's attenuation correction method. For the attenuation coefficient, 0.15 cm^−1^ was used based on the previously obtained pooled phantom data. A Ramachandran filter was used as the reconstruction filter.

## 3 Statistical Analyses

According to the NPI results, patients were divided into two groups: one with TH (DLB + TH) and the other without (DLB − TH). We used the Mann–Whitney *U* test to compare continuous or ordinal variables, and Fisher's exact test to compare the proportions of categorical variables between the groups. The threshold for significance was *p* < 0.05.

All statistical analyses were conducted using JMP 15 (JMP Statistical Discofery LLC, Cary, NC, US).

In cases where MRI could be collected, the difference in regional grey matter volume (rGMV) between DLB + TH and DLB − TH was examined with a voxel‐by‐voxel comparison using a two‐sample t‐test model in SPM. The total intracranial volume was inserted into the comparison as a nuisance covariate. The statistical threshold was set to an uncorrected *p* < 0.005; the extent threshold was set to 200 voxels.

In cases where SPECT images could be collected, three‐dimensional stereotactic surface projection (3D‐SSP) images were created using the Neurological Statistical Image Analysis Software (NEUROSTAT) [23]. Image analysis was performed using iSSP version 3.5, FALCON version 6.1.0.0 (Nihon Medi‐Physics Co. Ltd., Tokyo, Japan). In this process, the spatial distribution of abnormal rCBF was calculated for each SPECT datum using the age‐sex‐matched normal control database of The University of Osaka Hospital. The spatial distribution of abnormal rCBF was compared between the DLB + TH and DLB − TH groups by calculating two‐sample t‐statistic values (converted to Z) at each voxel using iSSP3.5_2tZ (Nihon Medi‐Physics Co. Ltd.). The statistical Z‐threshold was set to 2.

## 4 Results

### 4.1 Clinical Characteristics

Data of 147 patients who met the criteria for probable DLB were extracted from the database. Among them, two patients were excluded because of a lack of NPI data. Of the remaining 145 patients, 10 were assigned to the DLB + TH group and 135 to the DLB − TH group, according to the NPI results. Therefore, the prevalence of TH in patients with DLB was approximately 6.9%.

In the analyses of clinical features, there were no significant differences in age, sex, or cognitive function between the groups. The disease duration was significantly longer in the DLB + TH group (Table 1). The proportion of patients using cholinesterase inhibitors tended to be higher in the DLB + TH group; however, this difference was not statistically significant. There was no significant difference in the rate of antipsychotic drug use between the two groups. None of the patients in the DLB + TH group were prescribed anti‐parkinsonian drugs.

**TABLE 1:** Demographic data of patients with DLB.

Variables	DLB + TH (*n* = 10)		DLB − TH (*n* = 135)		*p* ^a^
	Mean	(SD)	Mean	(SD)	
Age (years)	81.6	(6.6)	78.3	(5.8)	0.189
Disease duration	6.0	(5.1)	2.96	(2.5)	0.049*
Education	12.1	(2.3)	11.7	(3.2)	0.810
MMSE	19.0	(6.6)	19.8	(5.3)	0.936
CDR	1.5	(0.9)	1.2	(0.7)	0.278

	Number	(Percent)	Number	(Percent)	*p* ^b^
Sex (males)	5	(50%)	66	(49%)	0.999
Using ChEI	6	(60%)	46	(34%)	0.168
Using AP^c^	4	(44%)	55	(43%)	0.999

Abbreviations: AP, antipsychotic drugs; CDR, clinical dementia rating; ChEI, cholinesterase inhibitors; DLB − TH, group of patients with DLB who did not have tactile hallucinations; DLB + TH, group of patients with DLB who had tactile hallucinations; DLB, dementia with Lewy bodies; MMSE, mini‐mental state examination; SD, standard deviation.

* *p* < 0.05.

^a^ Mann–Whitney *U* test.

^b^ Fisher's exact test.

^c^ One case of DLB + TH and eight cases of DLB − TH with missing data.

TH effects consist mostly of sensations of small objects such as insects, animals, strings, and hands touching, crawling, or clinging to the patient's skin. Three patients claimed that someone's hands crept into their underwear, night clothing, or blankets in a sexual context. Although all patients with TH complained of abnormal skin sensations, none of them developed delusional infestation, which is a conviction of being infested with small living or nonliving organisms.

**FIGURE 1:**
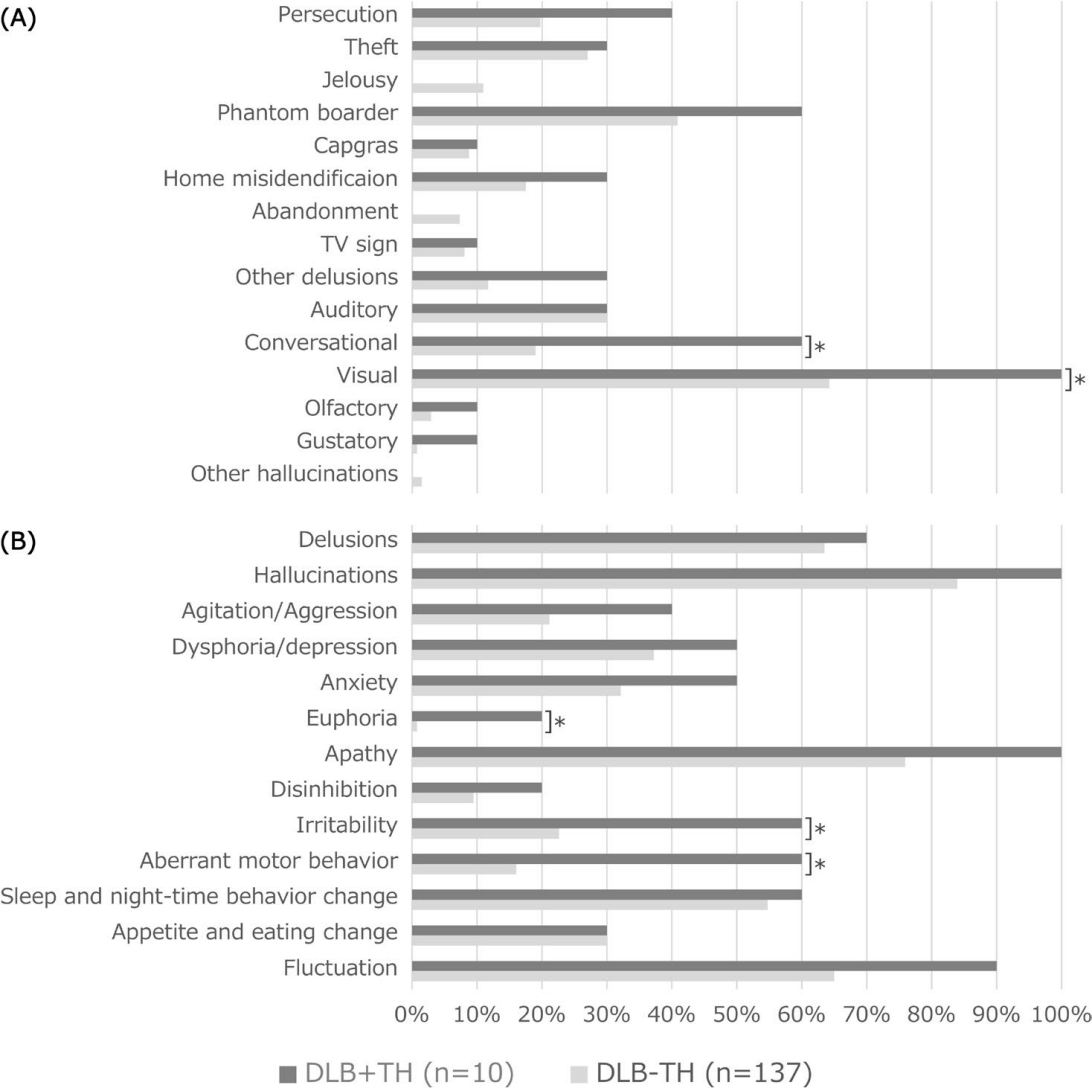
Rates for each type of delusions, hallucinations (A) and other neuropsychiatric symptoms (B).*p < 0.05 (Fisher's exact test). DLB, dementia with Lewy bodies; DLB + TH, group of patients with DLB who had tactile hallucinations; DLB − TH, group of patients with DLB who did not have tactile hallucinations.

The patients in the DLB + TH group tended to have more hallucinations than those in the DLB − TH group (Figure 1A). In particular, VH was observed in all patients in the DLB + TH group, and there were significantly more conversational monologue (talking to people who were not there) in the DLB + TH group. Furthermore, NPS other than hallucinations and delusions were observed more frequently in the DLB + TH group (Figure 1B). In particular, the rates of euphoria, irritability, and aberrant motor behavior were significantly higher in the DLB + TH group.

### 4.2 Neuroimaging Analysis

**FIGURE 2:**
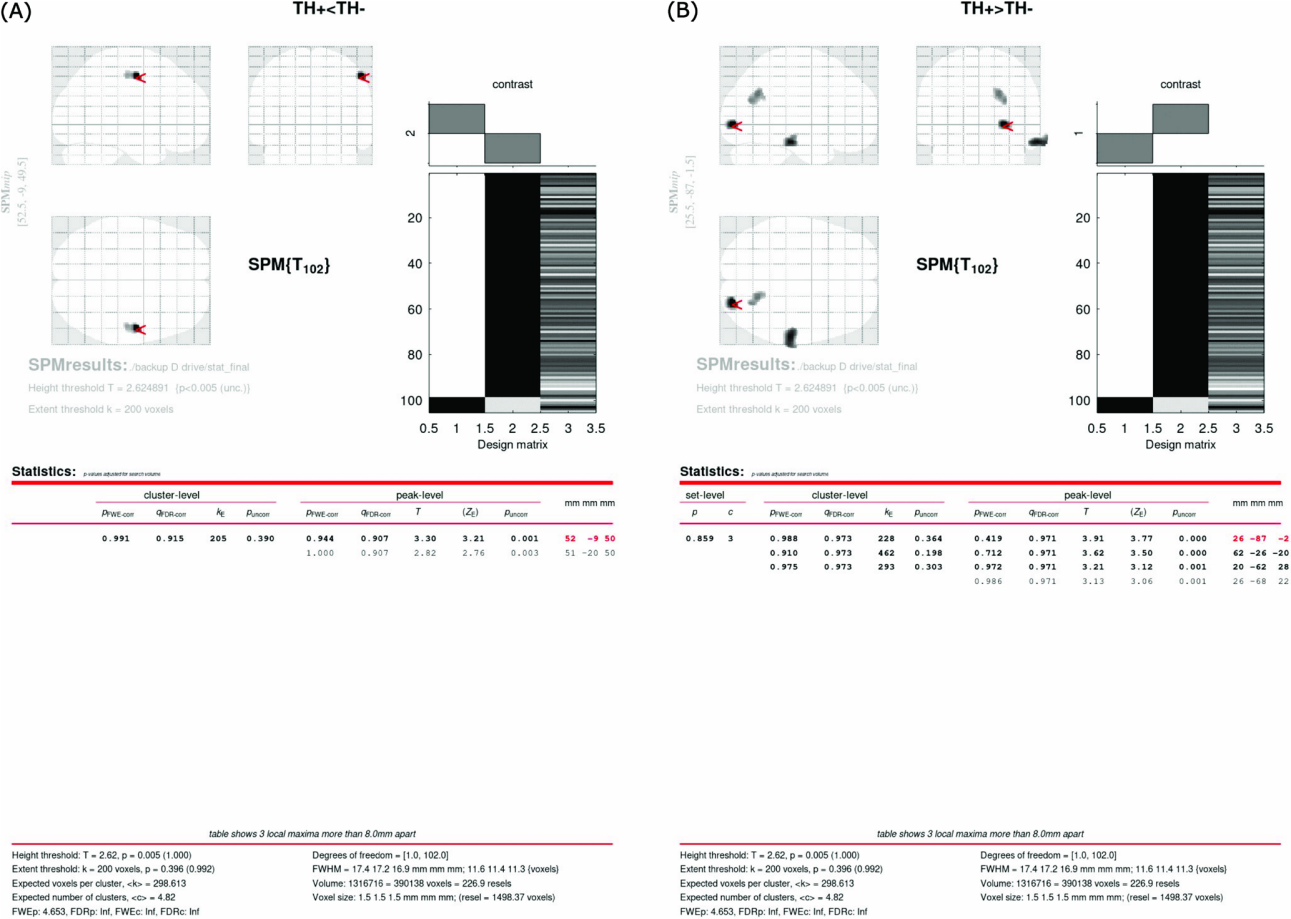
Brain regions with lower (A) and higher (B) grey matter volume in DLB + TH than in DLB − TH, statistical threshold at uncorrected *p* < 0.005 and extent threshold at 200 voxels. DLB, dementia with Lewy bodies; DLB + TH (TH+), group of patients with DLB who had tactile hallucinations; DLB − TH (TH−), group of patients with DLB who did not have tactile hallucinations. SPM, statistical parametric mapping.

In the MRI analysis, three patients were excluded from the DLB + TH group and 37 from the DLB − TH group because of the lack of 3D‐T1 images. We conducted an MRI analysis of 105 patients (seven in the DLB + TH group vs. 98 in the DLB − TH group). In the SPM analysis of MRI, rGMV of the upper part of the right postcentral gyrus was significantly lower in the DLB + TH group than in the DLB − TH group (Figure 2A). In contrast, rGMV of the lower part of the right parietal lobe, a part of the right occipital lobe, and a part of the right middle temporal gyrus was significantly higher in the DLB + TH group than in the DLB − TH group (Figure 2B).

**FIGURE 3:**
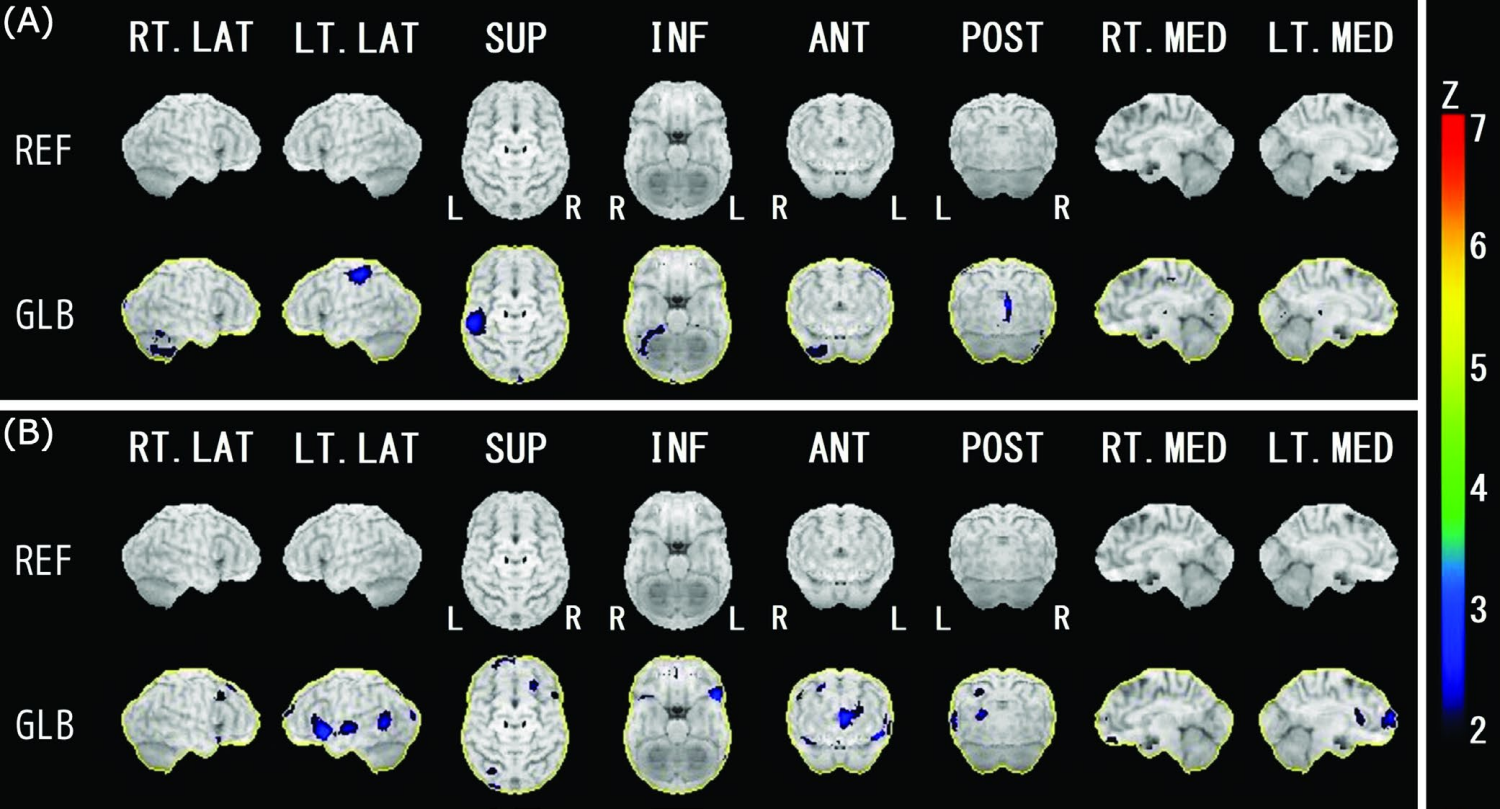
Brain regions with lower (A) and higher (B) cerebral blood flow in DLB + TH than in DLB − TH. Color coding represents the statistical significance (Z‐score). Data was normalized by whole brain average count (GLB). DLB, dementia with Lewy bodies; DLB + TH, patients with DLB who had tactile hallucinations; DLB − TH, patients with DLB who did not have tactile hallucinations; REF; anatomical reference map.

In SPECT images analysis, seven patients were excluded from the DLB + TH group and 34 from the DLB − TH group due to a lack of images. We analyzed the SPECT images of 104 patients (three in the DLB + TH group vs. 101 in the DLB − TH group). In one patient in the DLB − TH group, the data were imaged with Symbia Intevo; all other case data were imaged with Symbia T‐6. In the iSSP3.5_2tZ analysis, rCBF in the upper part of the left postcentral gyrus was significantly lower in the DLB + TH group than in the DLB − TH group (Figure 3A). In contrast, rCBF in a part of the left parietal lobe, a part of the left occipital lobe, and a part of the left superior temporal gyrus was significantly higher in the DLB + TH group than in the DLB − TH group. Furthermore, rCBF in the left inferior frontal gyrus, left frontal pole, and right superior/middle frontal gyrus was significantly higher in the DLB + TH group (Figure 3B).

**FIGURE 4:**
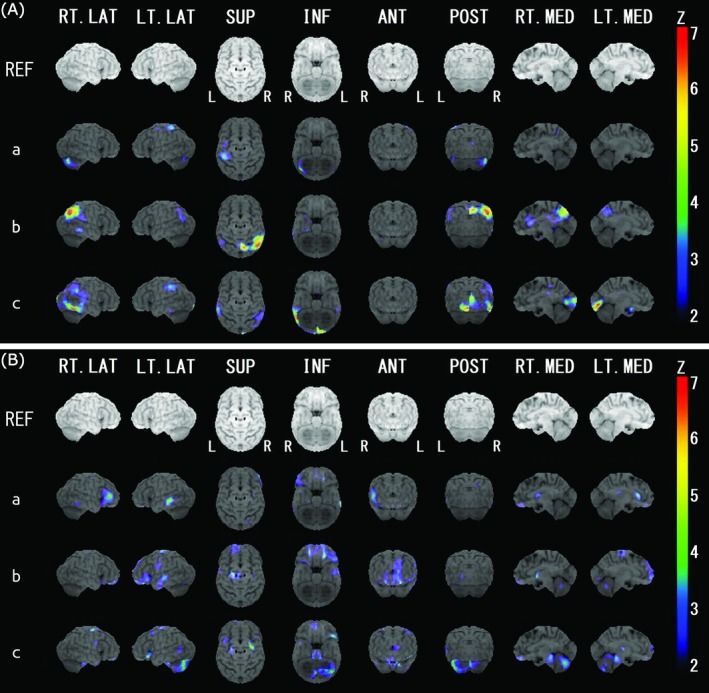
Brain regions with lower (A) and higher (B) cerebral blood flow in each DLB + TH case than in normal controls. Color coding represents the statistical significance (Z‐score). DLB, dementia with Lewy bodies; DLB + TH, patients with DLB who had tactile hallucinations; REF, anatomical reference map.

As there were only three patients in the DLB + TH group in the SPECT analysis, we examined the results from the analyses of the individual SPECT images of these three patients against age‐sex‐matched healthy controls in the iSSP (a, b and c in Figure 4A and 4B). Decreased rCBF was observed in the left postcentral gyrus in two patients (a, c) and in the right postcentral gyrus in one patient (b) compared to the normal control (Figure 4A). Two patients (b, c) had lower rCBF in the parieto‐temporo‐occipital region as well, which is consistent with DLB features. Increased rCBF was observed in the right lower frontal region (a) and in the left lower frontal region (b, c) (Figure 4B).

## 5 Discussion

In this study, we investigated the clinical characteristics and brain imaging features of patients with DLB presenting with TH. To our knowledge, this was the first study to explore the neural basis of TH in patients with DLB. The results indicated that TH prevalence in patients with DLB was approximately 7%; they tended to have a longer disease duration, had NPS other than TH, and their brain imaging showed atrophy and hypoperfusion in the upper part of the postcentral gyrus.

A previous comprehensive review of NPS in PD reported that the prevalence of TH in PD ranged from 0% to 22.5% [24], and a cross‐sectional comparative study of NPS in PD with or without dementia and DLB reported that TH prevalence was 0%, 1%, and 3%, respectively [25]. In the current study, TH prevalence was 6.9%, which is consistent with the results of previous studies.

There were no significant differences in demographic data between the two groups in our study, except for disease duration. The use of cholinesterase inhibitors appears to be low compared to the general clinical situation (60% in the DLB + TH group, 34% in the DLB‐TH group); this is due to the data from the time of the first consultation at our hospital, when DLB had not yet been diagnosed (DLB would be diagnosed based on the test results at that time). In DLB, neither age (at exam, at onset), sex, major depression, cognitive impairment, nor taking anti‐parkinsonian agents were reported to be associated with hallucinations [25]. In a previous case series of TH in PD, most patients with PD with TH had a long disease duration (mean > 11 years) and a high severity level, suggesting a link between advanced PD and TH presence [14]. Furthermore, in a prospective cohort study of patients with PD, the prevalence of TH increased over time, increasing from 20.25% at 1.5 years to 33.75% at 10 years of age [26]. Given the present results, the same may be true for DLB.

The characteristics of TH in PD are typically the sensation of animals or insects biting or crawling on the skin, or the sensation of liquid such as water or oil running or dripping from the skin according to some case reports [13, 14, 15] and a review of them [24]. The only previous study that reported a case series of TH in patients with DLB [16] also reported that their characteristics were very similar to those of patients with PD reported in the above studies. The present results are consistent with these previous papers as well in this respect. Additionally, in previous reports of PD, it was elaborated that hallucinations are accompanied by other types of hallucinations occurring simultaneously [14], and the hallucinations of different modalities interacted with one another [24]. Also in DLB, NPS other than hallucinations were seen more frequently in those with hallucinations than in those without [12]. These results are consistent with the characteristics of the DLB + TH group compared to the DLB − TH group in the present study.

In the present study, none of the patients had strong delusions associated with TH, such as delusional infestations or cenesthesia. Three quarters of patients with TH in PD were reported to retain the insight into the hallucinatory nature of the phenomenon despite their reality and did not have delusional infestations nor cenesthopathic hallucinations [14]. Considering these facts, not many patients with DLB seem develop strong delusions from TH. However, it was reported that some TH cases in PD or DLB exhibited delusional infestations or cenesthopathy [13, 14, 27] and led to extreme behavior such as burning the own body to exterminate insects attached to the body [17]. In DLB, even if patients saw a vision of an unfamiliar person in their home, VH could be determined by them unless they experienced auditory hallucinations; once the hallucination spoke, they were more likely to believe it existed [12]. There seems to be a tendency that hallucinations in one modality enhance the hallucinations in other modalities, making them more believable, for example “strengthening of VH by TH” [16]. However, even if the TH and VH of insects are experienced simultaneously, it does not necessarily result in delusional infestations. Delusional infestations seem to have another mechanism, which may be delusionality.

To the best of our knowledge, the first report of TH in a patient with PD was a drug‐induced case involving the use of pergolide and levodopa [13]. Another case report stated that TH in patients with PD was triggered by dopamine agonists [27], and a review indicated that the most important extrinsic psychotogenic factor in patients with PD is dopaminomimetic stimulation of the limbocortical dopaminergic receptors [28]. TH in these patients was accompanied by strong delusions; that is, they would exhibit characteristics of delusional infestations or cenesthopathy. Another review reported that hallucinations were not associated with the number and dose of anti‐parkinsonian agents in patients with PD or DLB [25]. Our results indicated that none of the patients with TH had been using anti‐parkinsonian drugs, and their use may be relevant, but not essential, for TH development.

The pathophysiology of TH and other types of hallucinations in DLB, as is PD, is uncertain. Some hypotheses have been proposed to explain the mechanisms of hallucinations. In a previous study that explored co‐occurring multi‐sensory hallucinations in patients with different conditions including PD and DLB [29], a “generative model of perception” [30] was introduced in which hallucinations may be understood. The hypotheses regarding hallucinations can be interpreted as: (1) impairment of the sensory center and release of symptoms, (2) psychosis or disturbance of consciousness, and (3) cognitive dysfunction. In the present study, brain imaging showed atrophy and hypoperfusion in the upper part of postcentral gyrus as well as higher rCBF in left inferior frontal gyrus in the DLB + TH group than the DLB − TH group, although tactile hallucinations were not associated with sleep disturbances, cognitive fluctuations, nor cognitive impairment. The results suggest that tactile hallucinations in the present patient group is related to the first hypothesis.

It is said that the VH seen in PD is similar to those described in Charles Bonnet syndrome associated with vision loss [24], long described as a release phenomenon, that is, removal of normal sensory impulses releases indigenous cerebral activity of the sensory system [31]. In a previous study that compared structural imaging studies of patients with VH in PD and DLB, atrophy of both primary and secondary visual cortex in the occipital lobe was found in each clinical condition [32]. Similarly, the auditory hallucinations in DLB are hypothesized as the auditory input to the primary auditory cortex is decreased in patients with hearing impairment; the reduced basal inhibition of the auditory association cortex might allow spontaneous activity [12].

The postcentral gyrus has long been considered the somatosensory cortex [33]. Although it is considered that there is no clear boundary with the motor cortex of the precentral gyrus and that they intermingle with each other [34], a recent somatosensory mapping study has confirmed the postcentral gyrus as the somatosensory cortex [35]. The presence and severity of skin sensation impairment were not examined in our study; however, our results that brain imagings of patients with DLB with TH showed atrophy and hypoperfusion in the upper part of the postcentral gyrus are consistent with the conditions of reduced primary input of sensory information in “release phenomena” hypothesis for TH. A functional MRI study reported that patients with PD with chronic VH responded to visual stimuli with greater frontal and subcortical activation and less visual cortical activation than non‐hallucinating participants with PD [36]. This is consistent with the relatively higher rCBF in the left inferior frontal gyrus in the DLB + TH group in the present study.

This study has several limitations. First, it was not possible to directly investigate internal psychiatric symptoms, such as hallucinations, in patients. Since hallucinations were assessed based on caregiver observations, the content of hallucinations tended to be biased toward those that were most memorable to the caregivers. This methodological issue may indicate that the rate of hallucinations is not as high as it is in reality. Second, the retrospective examination of case notes to identify any kind of symptoms is likely to have led to underreporting. However, in our institution, the NPS is assessed using the NPI‐plus for all patients suspected of having dementia; therefore, the impact of this limitation may have been minimized. Third, as there were some missing data on anti‐parkinsonian medication history, the statistical analyses of it could not be performed. However, it was confirmed that no anti‐parkinsonian drugs were prescribed for any of the DLB + TH group. There were also some missing data on antipsychotics; however, as the missing data were less than 10% and occurred completely at random, the statistical results were presented after excluding the missing data. Fourth, the small number of participants in the DLB + TH group had low statistical power, whereas the total number of participants with DLB was 145. Therefore, to extract features from the neuroimages of the DLB + TH group, it was necessary to use a looser threshold in image analyses. Finally, this was a cross‐sectional study. Although a relationship between TH and brain rGMV or rCBF was shown, a causal relationship is unclear.

In conclusion, the present study revealed that patients with DLB with TH often had hallucinations in other modalities and various NPS. In DLB, TH may be related to damage in the postcentral gyrus and preserved function in the frontal lobe, which may be consistent with the “release phenomena” hypothesis for hallucinations. The prevalence of TH in patients with DLB is low, and this study has many limitations. Further research is required to resolve these limitations.

## Ethics Statement

This study was approved by the Internal Review Board of The University of Osaka Graduate School of Medicine (No. 19117). The study conforms to the provisions of the Declaration of Helsinki (as revised in Brazil 2013).

## Consent

All the patients and their families agreed to an opt‐out clause for the use of data collected during common clinical examinations.

## Conflicts of Interest

Kyosuke Kakeda and Hideki Kanemoto received lecture fees from Sumitomo Pharma Co. Ltd. Hideki Kanemoto, Kenji Yoshiyama, Mamoru Hashimoto, and Manabu Ikeda received grants and personal fees from Eisai Inc. The other authors declare no conflicts of interest.

## Data Availability

The datasets used and/or analysed during the current study are available from the corresponding author upon reasonable request. The original contributions presented in the study are included in the article material; further inquiries can be directed to the corresponding author.
